# Pupillary responses to light are not affected by narrow irido-corneal angles

**DOI:** 10.1038/s41598-017-10303-3

**Published:** 2017-08-31

**Authors:** A. V. Rukmini, Raymond P. Najjar, Eray Atalay, Sourabh Sharma, Jing Zhan Lock, Mani Baskaran, Monisha Nongpiur, Joshua J. Gooley, Tin Aung, Dan Milea

**Affiliations:** 1 Singapore Eye Research Institute, Singapore, 169856 Singapore; 2 The Ophthalmology & Visual Sciences ACP (EYE-ACP), SingHealth and Duke-NUS, Singapore, 169857 Singapore; 3 Singapore National Eye Centre, Singapore, 168751 Singapore; 4 Program in Neuroscience and Behavioural Disorders, Duke-NUS Medical School, Singapore, 169857 Singapore; 5 Department of Physiology, Yong Loo Lin School of Medicine, National University of Singapore, Singapore, 117597 Singapore; 6 Department of Ophthalmology, Yong Loo Lin School of Medicine, National University of Singapore, Singapore, 119228 Singapore

## Abstract

Chromatic pupillometry is an emerging method for evaluating ocular health that relies upon the differential stimulation of rods, cones, and intrinsically photosensitive retinal ganglion cells (ipRGCs). Although it has been investigated in conditions affecting the outer or inner retina, there is a paucity of studies in conditions where the anterior chamber of the eye is affected. Primary angle closure suspects (PACS) are defined as eyes with narrow anterior chamber angles and intact retina. PACS patients are at risk of developing primary angle closure glaucoma and are prophylactically treated by performing laser peripheral iridotomy (LPI). Here we evaluated pupillary responses to monchromatic lights in 18 PACS before and after LPI, and compared the results with those of 36 age-matched controls who had gonioscopically open angles. Dose response curves for pupillary constriction were similar between PACS patients and controls (*p* = 0.98 for blue and 0.90 for red light) and within subjects pre- and post-LPI (*p* = 0.58 for blue and 0.20 for red light). Baseline-adjusted pupillary constriction responses to blue and red lights were similar in controls and PACS, and not altered after LPI. Our findings suggest that narrow irido-corneal angles and LPI do not influence pupillary responses in PACS.

## Introduction

Pupillary responses to light are dependent on the integrity of the retina as well as intact afferent and efferent neural pathways^1–3^. These responses are controlled by retinal rods, cones, and the melanopsin containing intrinsically photosensitive retinal ganglion cells (ipRGCs)^1, 4, 5^. Since each of these photoreceptors responds to lights of different wavelengths and intensities, chromatic pupillometry provides a unique way to detect deficits in the functioning of these photoreceptors^1, 4–6^. We have previously developed a ramp-up light exposure protocol, wherein rods, cones and ipRGCs are sequentially stimulated^7^ and evaluated pupillary responses in healthy subjects^8^, patients with primary open angle glaucoma (POAG)^7, 9^, and cataract^10^. While retinal deficits, as seen in POAG, lead to alterations in pupillary responses^7, 9^, anatomical factors of the anterior segment can also affect the pupillary light responses in healthy subjects^8^.

Primary angle closure suspect (PACS) eyes are defined as eyes with occludable anterior chamber angles, without peripheral anterior synechiae (PAS) or raised intra ocular pressure (IOP), and associated with normal optic discs and normal visual fields^11^. These eyes are said to have narrow irido-corneal angles and at risk for developing primary angle closure glaucoma (PACG)^12, 13^, which is an important cause of ocular morbidity in Asia, especially with the rapidly ageing population^14^. PACG is most often an insidious/ asymptomatic disease, which has a higher risk for blindness than primary open angle glaucoma^15, 16^. It is therefore essential to identify patients at an early stage of angle closure disease spectrum, as PACS, in order to prevent its progression. Prophylactic treatment of PACS by laser peripheral iridotomy (LPI) is commonly performed to prevent progression to PACG, by preventing pupillary block, which is the predominant mechanism involved in angle closure^17–19^. LPI widens the irido-corneal angle and equilibrates the pressure difference between the anterior and posterior chambers by creating a small opening in the peripheral iris^20, 21^.

We, and others, have shown that pupillary responses to high-irradiance blue light correlated with severity in POAG^7, 9, 22^. However, to date, chromatic pupillometry has not been evaluated in patients with narrow angles or patients with angle closure glaucoma. Recent studies have shown differences in pupil reactivity in PACG compared to controls^23, 24^ and that healthy subjects with thinner irides show increased constriction amplitude to blue and red lights^8^. The aim of this study was to evaluate if narrow irido-corneal angles in PACS affect baseline pupil diameter and pupillary responses to chromatic pupillometry in the absence of glaucomatous optic neuropathy, thus refining the role of pupillometry in glaucoma screening. Furthermore, we assessed whether LPI alters chromatic pupillometry parameters in PACS through mechanical changes in the iris.

## Results

### Subject characteristics

A total of 54 subjects, (18 PACS and 36 healthy controls) participated in the study. Participants were predominantly females and of Chinese descent (Table 1).

**Table 1:** Demographic characteristics of the subjects.

Subject group	n	Number of males (%)	Number of Chinese subjects (%)	Age in years (mean ± SD (Range))
PACS	18	6 (33.3%)	18 (100%)	66.72 ± 7.1 (55–79)
Control	36	15 (41.7%)	32 (88.9%)	66.64 ± 5.6 (55–75)

### Baseline ophthalmic measures

Baseline ophthalmic parameters in PACS before LPI and control participants are presented in Table 2. The baseline intra-ocular pressure, cup-disc ratio, visual field mean deviation, and average RNFL thickness were similar between groups. In contrast, PACS patients exhibited a significantly shorter axial length, shorter mean anterior chamber depth, and hyperopic refraction compared with control subjects.

**Table 2:** Ophthalmic examination results in PACS and control subjects.

Test parameter	PACS (mean ± SD)	Control (mean ± SD)	p
Intra-ocular pressure (mmHg)	14.67 ± 2.5	15.0 ± 2.5	0.64
Cup-disc ratio	0.48 ± 0.1	0.44 ± 0.1	0.07
Axial length (mm)	23.06 ± 0.8	24.00 ± 1.0	**<0**.**001**
Anterior chamber depth (mm)	2.59 ± 0.3	2.98 ± 0.3	**<0**.**001**
Spherical equivalent (D)	1.15 ± 1.0	−0.29 ± 2.0	**<0**.**001**
Visual field mean deviation (dB)	−2.27 ± 4.1	−1.83 ± 1.9	0.67
Average RNFL thickness (µm)	94.94 ± 10.8	93.31 ± 8.8	0.58

### Baseline pupil diameter

**Figure 1:**
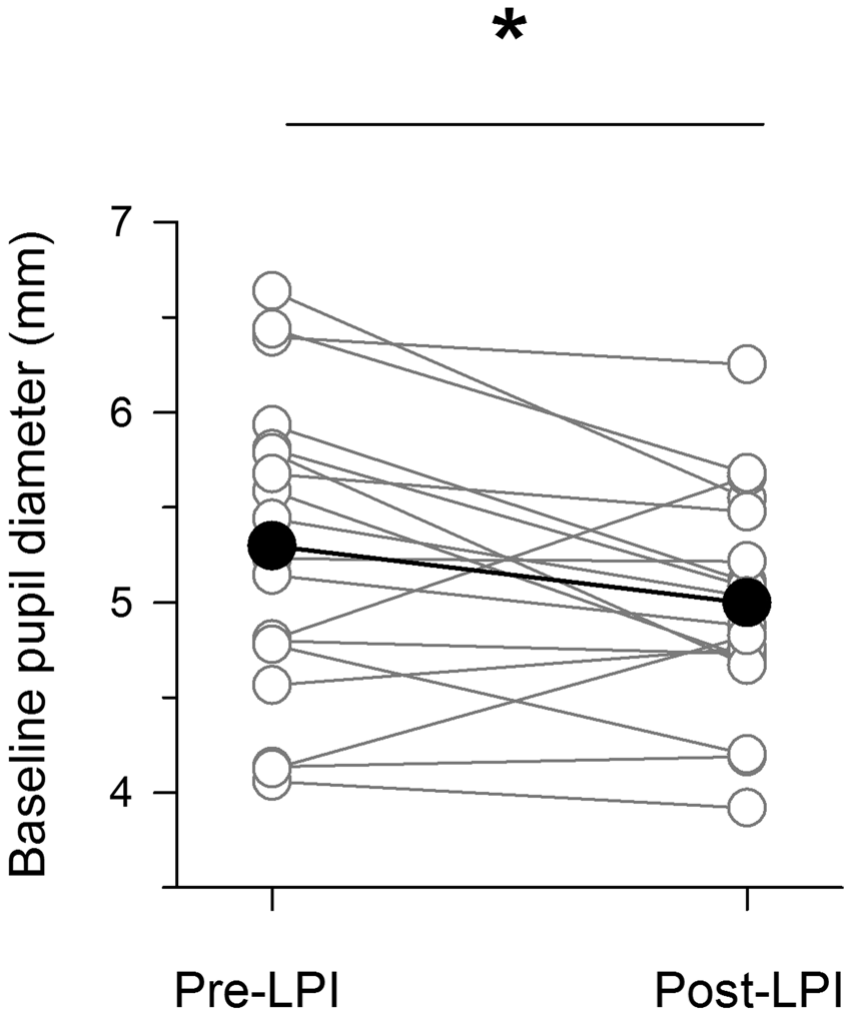
Average baseline pupil diameter decrease after LPI in PACS. Individual baseline pupil diameters pre- and post-LPI are plotted as grey empty circles connected by grey lines. The average baseline pupil diameters are represented as black filled circles connected by black lines. There is a significant reduction in baseline pupil diameter after LPI in PACS (p < 0.05).

Baseline pupil diameter did not differ between control subjects (5.56 ± 1.0mm) and pre-LPI PACS patients (5.30 ± 0.8mm) (*p* = 0.31). However, the average pupil diameter was significantly lower post LPI in PACS (5.00 ± 0.6mm), when compared to control subjects (*p* < 0.05) (Table 3). Following LPI, 14 out of the 18 PACS patients exhibited a smaller baseline pupil diameter, with the diameter decreasing by an average of 0.30 ± 0.56mm (*p* < 0.05) (Fig. 1).

**Table 3:** Parameters derived from chromatic pupillometry.

Experimental parameter	Controls	Pre-LPI PACS	Post-LPI PACS
Mean baseline pupil size (mm)**	5.56 ± 1.0	5.30 ± 0.8	5.00 ± 0.6*
Threshold of constriction to blue light (log photons cm^−2^ s^−1^)**	11.68 ± 1.1	11.00 ± 1.1*	11.64 + 0.9
Threshold of constriction to red light (log photons cm^−2^ s^−1^)	11.65 ± 0.8	11.17 ± 1.0	11.35 ± 1.2
Redilation latency after blue light offset (s)	20.40 ± 8.4	20.75 ± 9.2	22.81 ± 8.3
Redilation latency after red light offset (s)	27.51 ± 15.6	31.22 ± 15.7	28.92 ± 17.3

*p < 0.05 PACS patients have significantly lower value of the parameter.

**p < 0.05 within subject, paired tests.

### Chromatic pupillometry in PACS (pre-LPI) compared to controls

**Figure 2:**
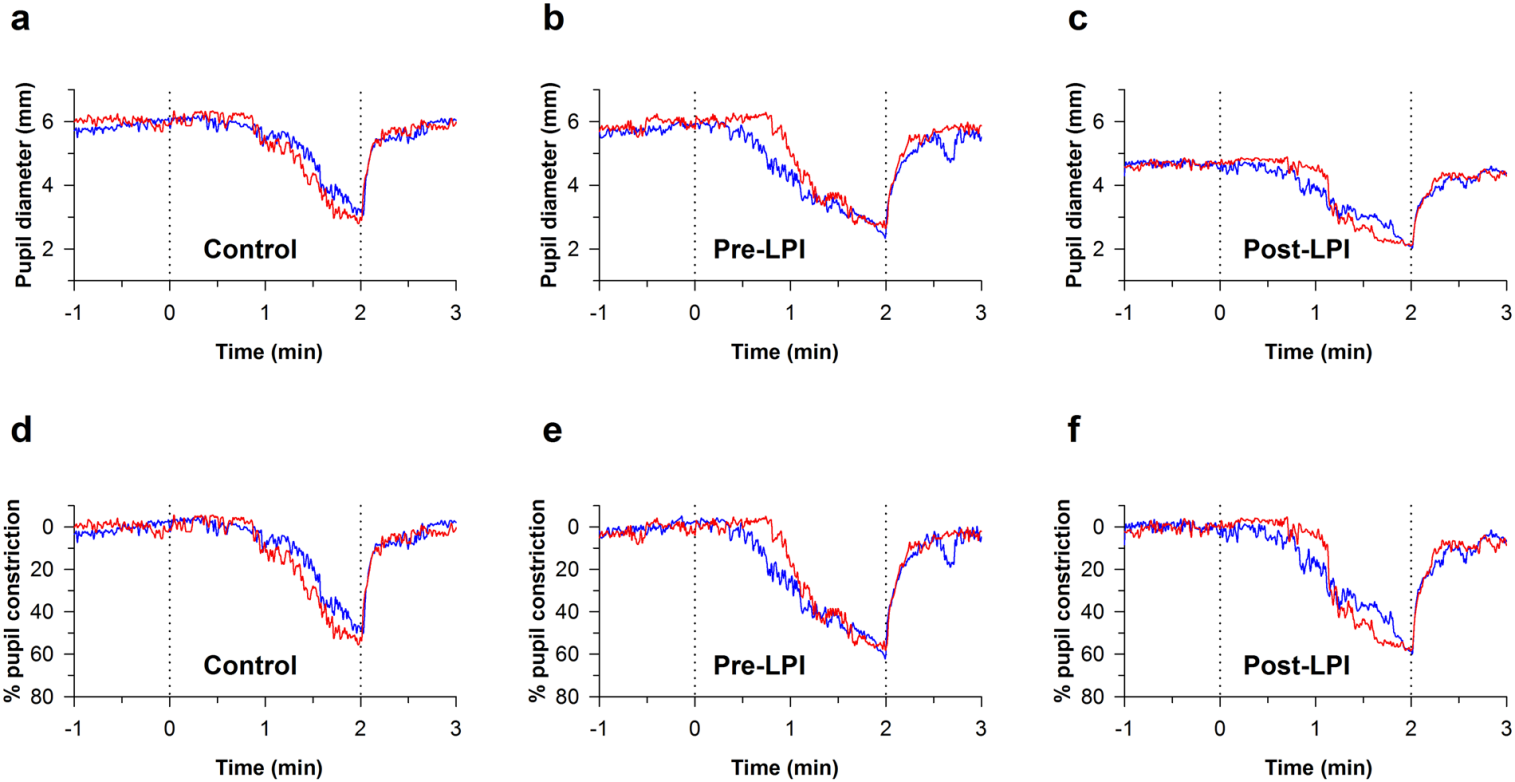
Representative pupillary constriction responses to blue and red lights in a control subject and in a PACS patient pre- and post-LPI. Representative absolute pupillary constriction responses in a control (**a**) and PACS patient pre- (**b**) and post- (**c**) LPI. Note the reduction in baseline pupil size from 5.8 to 4.7mm in the PACS patient following LPI (**c**). Such a reduction in baseline pupil size biases the qualitative consideration of the amplitude of pupillary responses to light. When the same profiles are corrected for baseline pupil size (**d**,**e**,**f**), pupillary constriction amplitude no longer appears to be reduced in the PACS patient following LPI (**f**).

**Figure 3:**
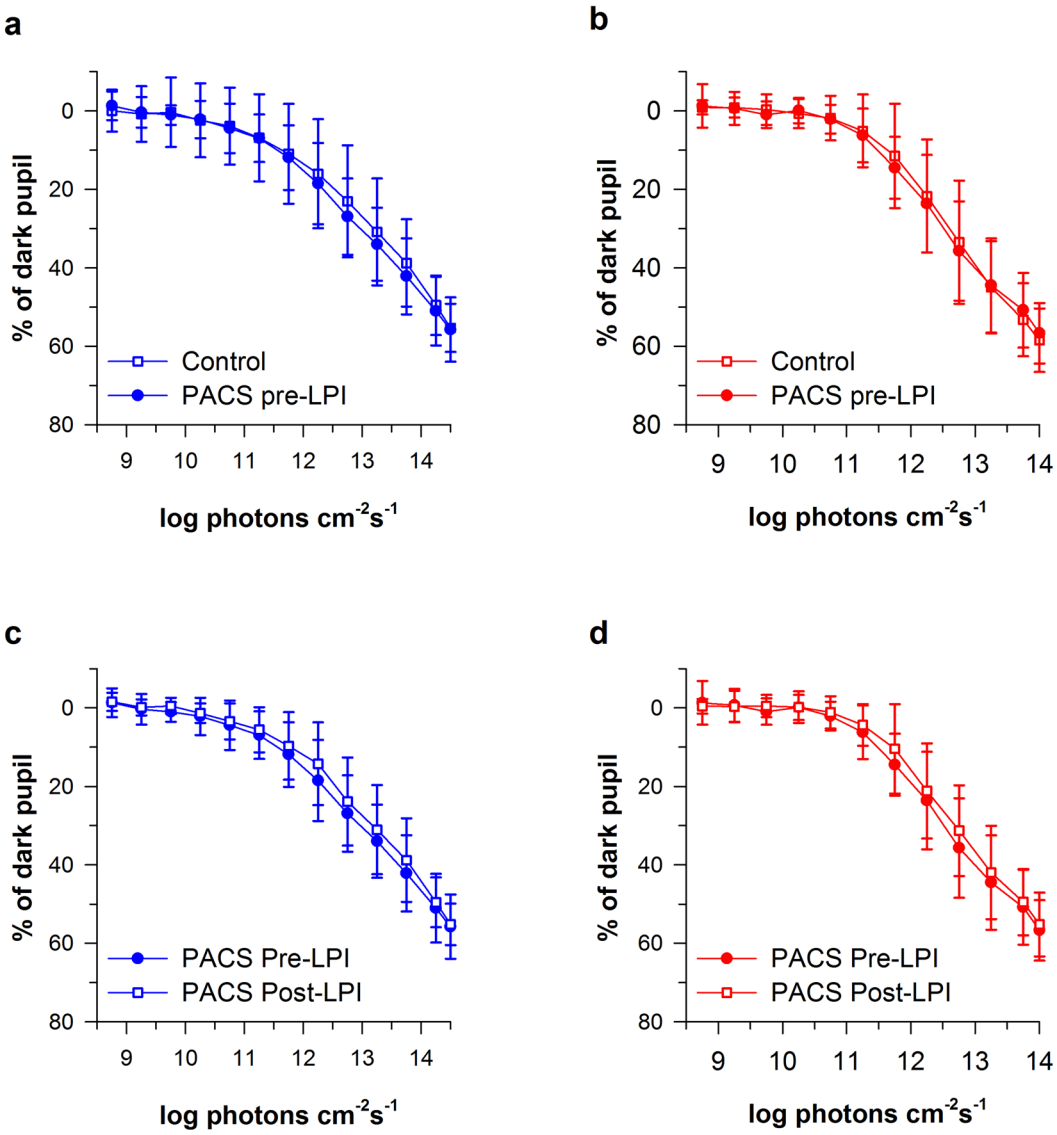
Pupillary constriction amplitude remains unchanged after LPI. Panels a and b show average pupillary constriction responses in Controls (open squares), and PACS subjects pre-LPI (filled circles). Panels c and d show average pupillary constriction responses in PACS subjects pre-LPI (filled circles) and post-LPI (open squares) exposed to blue light (469 nm, Panels a and c) and red light (631 nm, Panels b and d) stimuli. Data are presented as average ± SD.

Pupil size decreased in a dose dependent manner as a function of the gradually increasing blue and red lights in both controls (F_12,35_ = 318.59, p < 0.001 for blue light and F_11,35_ = 385.84, p < 0.001 for red light) and PACS patients pre-LPI (F_12,17_ = 260.68, p < 0.001 for blue light and F_11,17_ = 209.73, p < 0.001 for red light) (Figs 2a,b,d,e and 3a,b). There was no difference in the baseline-adjusted amplitudes of pupillary constriction between controls and PACS patients (pre- LPI) for either blue (*p* = 0.98), or red light stimuli (*p* = 0.90) (Fig. 3a,b). The threshold of constriction (defined as irradiance at which the pupil constricted by 10%) response was lower in PACS subjects pre-LPI when compared to controls (p < 0.05). Redilation latency (defined as the time taken for the pupil to reach to 90% of baseline after light offset) was not significantly different between the groups for both blue and red lights (Table 3).

### Chromatic pupillometry in PACS post-LPI compared to pre-LPI

Similar to controls and PACS pre-LPI, pupil size decreased with gradually increasing blue and red lights in PACS post-LPI (F_12,17_ = 260.36, p < 0.001 for blue light and F_11,17_ = 238.23, p < 0.001 for red light). The dynamics of pupillary constriction under blue (*p* = 0.58) and red (*p* = 0.20) lights remained unaltered in PACS patients post-LPI compared to constriction responses pre-LPI (Figs 2b–f and 3c,d). The threshold of constriction response increased significantly after LPI (*p* < 0.01), but redilation latency was not significantly different post-LPI for both blue and red lights (Table 3).

## Discussion

The main finding in our study is that the baseline-adjusted pupillary constriction profiles in PACS patients are not different from those of control subjects, indicating that anterior segment morphology itself does not alter light induced pupillary constriction responses. In addition, the constriction profiles remain unaltered in PACS following LPI, despite a significant reduction in baseline pupil diameter.

Spectral sensitivity of pupil constriction responses has been extensively investigated^25–28^. Melanopsin containing intrinsically photosensitive retinal ganglion cells (ipRGCs), rods and cones, are all involved in pupillary constriction responses^1, 4, 5^. Using our novel ramp-up light stimuli, it was possible to construct dose-response curves to light over a short time interval, across a wide range of irradiance levels, examining rod -cone and ipRGC activation using different wavelengths of light. Chromatic pupillometry has been increasingly employed for exploring various ocular conditions in humans associated with damage either to the photoreceptor layer or to ipRGCs. These include diabetic retinopathy^29^, age related macular degeneration^30^, ischemic optic neuropathy^31^, hereditary optic neuropathies^28^, and glaucoma^7, 32–34^. In this context, finding no difference in pupillary constriction responses despite differences in baseline pupillary size indicates that the influence of narrow angles and other biomechanical and dynamic iris dependent factors in PACS subjects might not alter pupillary responses substantially.

While our findings suggest that pupillary constriction responses do not differ between PACS and control subjects, a previous study found that the amplitude of pupillary constriction was reduced in subjects with shallow anterior chambers^35^. However, in that study, pupillary responses were not adjusted for the size of the baseline pupil, which was also shown to be different. In addition, only polychromatic white light was used to illuminate the pupil. Given the variations in baseline pupillary diameter, as well as the finding that LPI leads to a decrease in pupillary diameter in darkness, it is possible that differences in pupillary constriction reported in previous work are explained by the procedures used to analyse the data (i.e., without adjusting for baseline pupil diameter) as well as the usage of polychromatic light. In that same study, patients with acute primary angle closure (APAC) were also evaluated pre- and post-LPI^35^. There was no difference between baseline pupil diameter pre- and post-LPI, and consequently, there was no difference in the amplitude of constriction pre- and post-LPI, similar to our findings, indicating that LPI itself might not lead to differences in pupillary constriction responses.

In our study, there was no difference in the average baseline pupil size between control subjects and PACS patients pre-LPI. We also show that the absolute pupil diameter in darkness was reduced on average in PACS patients after LPI. This reduction in baseline pupil size can be due to a potential damage to the dilator pupillae during LPI. To our knowledge, however, such damage has not been directly implicated in the reduction in pupil diameter following LPI. Previous studies have reported conflicting data regarding the effect of LPI on baseline pupil diameter. While some studies have shown no effect of LPI on baseline pupil size^35–38^, others have shown decreased baseline pupil size post-LPI^39, 40^. In a recent study, pupil diameter (as measured by AS-OCT) is shown to be smaller after LPI in PACS eyes (4.67 ± 1.03mm vs 4.37 ± 1.03mm, one year post-LPI, p < 0.001, among 180 PACS subjects as part of a randomised controlled trial, unpublished data). In our subjects, pupillometry was performed 2 to 8 weeks after LPI. Pupil measurements in the other studies took place at various time intervals after LPI ranging up to several months. In addition, in our study, the patients were seated in a dark room, and the baseline pupil measurement was performed using a rigorous pupillometry protocol. This served to minimise sources of variance that can affect baseline pupil diameter, including background light.

Previous studies in PACG suggest that anatomical and/or biomechanical properties of the iris may influence the pupil dynamics^24, 37^. Iris bowing was suggested to be an important biometric parameter associated with dark–light changes in closed angle eyes^41^. It is also known that changes in iris area and volume in response to illumination differ between open and closed-angle eyes^42^. Using *in-vivo* ASOCT videography analysis, Zheng and colleagues reported a reduced acceleration of pupillary constriction and iris stretch in eyes with PACS compared to eyes with open angles, post-LPI^24, 37, 40^. In our study, using a ramp-up light exposure protocol, we did not note any differences in the slope of pupillary constriction responses. Zheng and colleagues also demonstrated that the speed of pupil constriction to white light increases in primary angle closure patients (more than 60% of who were diagnosed with PACS) after LPI^37^. In a previous study using chromatic pupillometry, we found that thicker irides reduce the constriction amplitude of the pupil, to blue and red lights^8^. These findings raise the possibility that the iris morphology plays a role in modifying the pupil responses. In this study, we did not study anatomical and biomechanical anterior segment parameters pre- and post-LPI. Nevertheless, while there may be a correlation between the anterior segment anatomy and pupillary constriction response, the effect is possibly not strong enough to drive differences as observed in our study.

The current study is limited by the small number of participants. Nevertheless, because the strict inclusion and exclusion criteria implemented in the study, we significantly reduced sources of variance (e.g. cataract status^10, 43^, age^10^, etc.) within and between groups. Also, it is important to test our protocol in other population cohorts, to resolve any possible ethnicity bias, as our study included predominantly ethnic Chinese subjects. It remains to be elucidated if pupillometry can detect PACS with a higher risk of progressing to PACG, either in the presence or absence of LPI, and this possibility should be explored in future research.

Angle closure is a major problem in Asian populations^44, 45^. These results suggest that in the absence of retinal changes, narrow angles alone, either in the presence or absence of LPI, do not influence pupillary constriction responses. Hence, narrow angles and/or LPI do not appear to preclude the use of chromatic pupillometry as a screening tool for retinal deficits. Similarly, in previous studies, we have shown that mild to moderate cataract does not influence pupillary light responses^10^. Thus, the current study adds to the corpus of knowledge that can be used in the future to detect functional deficits in retinal function.

## Methods

In this cross-sectional study, healthy control and PACS subjects, 50 years or older, were recruited from clinics at the Singapore National Eye Centre, Singapore. All subjects underwent a comprehensive ophthalmic examination, and demographic information was collected using interviewer-administered questionnaires.

Ophthalmic evaluation consisted of tests for visual acuity, automated refraction to assess the spherical equivalent (non-contact Auto Kerato-Refracto-Tonometer TRK-1P, Topcon), axial length and anterior chamber depth using IOLMaster (Carl Zeiss Meditec, Jena, Germany), slit-lamp examination, intraocular pressure measurement by Goldmann applanation tonometry, gonioscopy (performed in the dark using a Goldmann 2-mirror lens at high magnification ×16), clinical evaluation of cup-disc ratios. Standard automated perimetry (Humphrey Visual Field [HVF] Analyser II model 750; Carl Zeiss Meditec, Dublin, CA) and Optical Coherence Tomography (Cirrus HD-OCT, Carl Zeiss Meditec, Dublin, CA) were performed either on the day of chromatic pupillometry testing or taken within the preceding 8 months. Subjects were excluded if they had best corrected visual acuity of ≤6/15 in the study eye, refractive error ≥  ± 6.0 DSph or ≥3.0 DCyl, presence of ocular disease, previous intraocular injury or intraocular surgery (cataract extraction and lens replacement, previous LPI), or clinical features compatible with glaucoma or retinal diseases. Additional exclusion criteria included significant nuclear sclerosis of more than grade 2 severity on the Lens Opacification Classification System (LOCS) III (20). Patients suffering from neurological diseases or using ophthalmic drops which could affect light sensitivity or pupil size were also excluded from the study. The study eye for pupillometry was selected by a study team member, after evaluation of the inclusion and exclusion criteria. In case both eyes fulfilled the inclusion criteria, the study eye was randomly chosen.

The study was approved by the SingHealth Centralised Institutional Review Board and was performed in accordance with the tenets of the Declaration of Helsinki. Written informed consent was obtained from all subjects before enrolment.

### Primary angle closure suspect (PACS)

PACS was defined as eyes in which the posterior trabecular meshwork was not visible for at least 180 degrees on non-indentation gonioscopy (narrow angles) with the eye in the primary position, without peripheral anterior synechiae (PAS), or raised intraocular pressure (IOP, greater than 21 mm Hg), and without glaucomatous optic neuropathy or visual field defects^11^. LPI (Sequential Argon: Nd YAG) was performed between the 10-o’clock to the 2-o’clock positions, in the superior region of the iris, in all the included PACS patients. Pupillometric parameters were collected in the study eye pre-LPI (baseline assessment) and 2 to 8 weeks post-LPI in PACS. Post LPI medications including steroid eye drops were discontinued one week after LPI.

### Control subjects

Control subjects were defined as having normal ophthalmic examination and no past ophthalmic history, as well as no family history of glaucoma. Inclusion criteria were intraocular pressure ≤ 21 mm Hg with open angles, healthy optic nerves, normal visual fields, no previous intra-ocular surgery, and no family history of glaucoma. These subjects were age-matched and, on average, were similar in cataract status to the PACS patients, to control for any differences in pupillary responses due to ageing or cataract^10^.

### Chromatic Pupillometry testing

**Figure 4:**
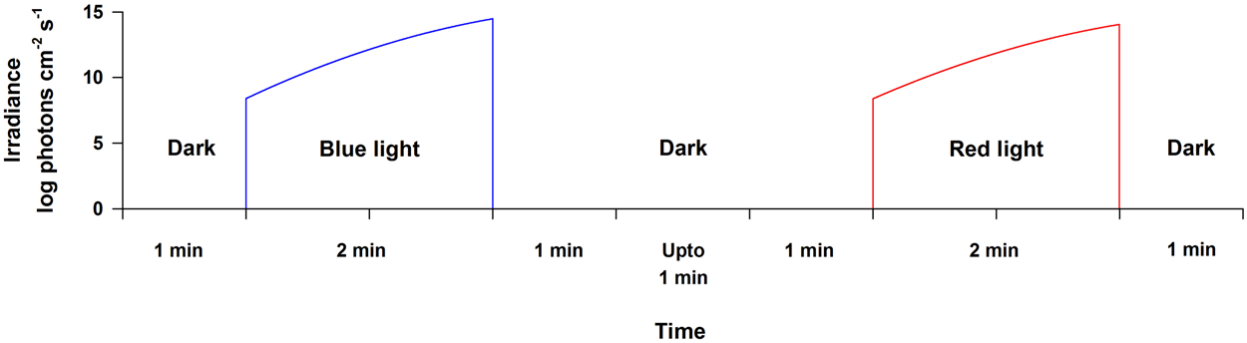
Light exposure protocol during the chromatic pupillometry procedure. Participants were exposed to two minutes of logarithmically increasing intensity of blue light followed by exposure to red light and pupillary responses to both lights were recorded in each subject. Each exposure to light was preceded and followed by one minute of darkness. There was a gap of up to one minute of darkness between the blue and red light exposures.

Chromatic pupillometry was performed in the PACS patients both pre- and post-LPI and in the control subjects. The details of the pupillometry set-up used here have been described elsewhere^7^. Briefly, direct pupillary light responses were studied in one eye in all subjects, with the other eye occluded by an eye patch. Light stimuli were administered using light emitting diodes (LEDs, Nichia Corporation, Tokushima, Japan), fixed on top of a modified Ganzfeld dome (Labsphere, Inc., North Sutton, NH) ensuring homogenous illumination across the visual field. Initial dark-adaptation was achieved during 1 min, while the patient was seated in darkness with his head fixed on a chin rest, while the pupil diameter was recorded using an infrared pupillometer (ETL-100H Pupillometry Lab; ISCAN Inc., Woburn, MA). The light stimulus was administered over 2 minutes in gradually increasing intensities of blue light (462 nm, 8.5 to 14.5 log photons cm^−2^s^−1^, first exposure) and red light (638 nm, 8.5 to 14.0 log photons cm^−2^s^−1^, second exposure) (Fig. 4). Each light exposure was followed by a 1-minute period of darkness, during which the subject remained seated in the dark room. This ramp-up light protocol aimed to activate rods, cones, and the intrinsic melanopsin-dependent response sequentially.

### Data analysis and Statistics

For each light exposure, pupil diameter measurements were processed using the Octave computing language^46^. After removal of blink artefacts, pupil size changes were normalised to baseline, calculated as the median pupil diameter in the 30 seconds before light onset. Parameters such as baseline pupil diameter, threshold of constriction (irradiance at which the pupil constricted by 10%), amplitude of pupillary constriction during the light exposure, and redilation latency (time until return to baseline) were analysed. Pupillary constriction as a function of light was binned by taking the median pupillary constriction response in 0.5 log unit bins from 8.5 to 14.5 (blue) or 14 (red) log photons cm^−2^s^−1^, resulting in 13 data points for blue light and 12 for red light exposure respectively. Threshold of pupillary constriction was defined as the irradiance of light at which pupillary constriction was 10% less than the baseline, and redilation latency was defined as the time taken for the pupil to reach to 90% of baseline after light offset. Redilation latency was corrected for both baseline pupil size and maximum constriction amplitude.

To study differences between PACS patients and controls, independent sample t-tests were used to compare baseline pupil size and other pupillometric measures collected during the chromatic pupillometry procedure. General linear model (GLM) analyses, with irradiance and group as factors, were used to compare pupillary dose response curves. For comparisons in PACS patients before and after LPI, paired student’s t-test and a two-way repeated measure ANOVA, with irradiance and group as factors were used. Pairwise multiple comparison procedures were performed using the Holm-Sidak method if the test reached statistical significance. In all cases, p < 0.05 was considered statistically significant. Statistical analyses were performed using SigmaPlot software (SigmaPlot 12.0, Systat Software, Inc., San Jose, CA). Data are presented as mean ± standard deviation, unless stated otherwise. Data used for the analyses (eliminating identifying information) can be made available on request.
